# Effects of Modified Tobacco Risk Products with Claims and Nicotine Features on Perceptions among Racial and Ethnic Groups

**DOI:** 10.3390/ijerph20156454

**Published:** 2023-07-27

**Authors:** Teresa DeAtley, Andrea C. Johnson, Matthew D. Stone, Janet Audrain-McGovern, Melissa Mercincavage, Andrew A. Strasser

**Affiliations:** 1 Department of Psychiatry and Tobacco Center of Regulatory Science, Perelman School of Medicine, University of Pennsylvania, Philadelphia, PA 19104, USA; andrea.johnson1@pennmedicine.upenn.edu (A.C.J.); audrain@pennmedicine.upenn.edu (J.A.-M.); melmer@pennmedicine.upenn.edu (M.M.); strasse3@pennmedicine.upenn.edu (A.A.S.); 2 Herbert Wertheim School of Public Health and Human Longevity Science, University of California, San Diego, CA 92093, USA; m3stone@health.ucsd.edu

**Keywords:** modified risk tobacco products, risk, harm, quitting, addictiveness, race, ethnicity

## Abstract

Research suggests consumers may misunderstand modified risk tobacco product (MRTP) claims. We examined the effects of nicotine content across four tobacco products with and without MRTP claims among a racially and ethnically diverse sample of adults who do and do not smoke. Adults (*n* = 1484) aged 21–65 completed an online experiment using a 2 × 2 × 4 mixed factorial design to examine the effects of tobacco product (Classic White Snus, IQOS, JUUL e-cigarette, and VLN cigarette) and nicotine content (high vs. low) stratified by MRTP claim (present vs. absent) across four outcomes: (1) likely to try (2) serious disease if used regularly, (3) least addictive, and (4) ease of quitting smoking. Not including an MRTP claim resulted in an increased likelihood of trying a product, decreased concern of serious disease, lower perceived addictiveness, and increased ease of quitting smoking. Participants selected low nicotine IQOS without a claim as the least likely to cause serious disease. Low nicotine JUUL, without a claim, was selected as least addictive and most likely to facilitate quitting. Intentions to try were highest for low nicotine JUUL. Participants selected low-nicotine products as less addictive than high nicotine products. Regulatory efforts should consider how MRTP claims interact with different product characteristics. Subtle differences exist across outcomes between racial and ethnic groups, which indicates that further research is warranted.

## 1. Introduction

The 2009 Family Smoking Prevention and Tobacco Control Act grants the Food and Drug Administration (FDA) the authority to regulate the marketing, distribution, and manufacturing of tobacco products. The purview of the FDA’s regulatory authority has since expanded to include the classification of tobacco products determined to have modified risk after passing an in-depth scientific review process [1]. Products that receive either a modified risk or exposure order can be marketed using claims of lower risk of disease or posing less exposure to harmful constituents [1]. To date, 16 modified risk orders have been granted (nine smokeless tobacco products, one heated tobacco product (HTPs), and six cigarettes (heat sticks and combustible cigarettes) [2]. Research has shown that consumers misunderstand aspects of modified risk tobacco product (MRTP) claims, such as believability [3] and perceived product risk reduction [4]. Existing misperceptions about nicotine content and perceived health risks for non-combustible tobacco products such as e-cigarettes [5] may, in part, explain why MRTP claims are misinterpreted. E-cigarette manufacturers were only recently required to include a warning label stating that the product contains nicotine as a result of the FDA’s 2016 deeming rule [6]. Researchers have tested a variety of e-cigarette warning statements [7], including those of modified risk [8], as e-cigarettes are increasingly regarded as being lower in risk to combustible cigarettes [9,10]. 

In a systematic review focused on nicotine reduction, Glasser and Berman identified two key populations in which nicotine knowledge was inadequate [11]. Specifically, foreign-born individuals were found to have greater misperceptions about cigarettes advertised as low nicotine [12] relative to U.S.-born individuals, and racial and ethnic minorities were more likely to believe that nicotine causes cancer relative to Non-Latine White individuals [13]. Research on nicotine beliefs across different demographic subgroups is sparse [14], and further exploration of the heterogeneity that may exist between populations is needed. Nicotine content varies between reduced-risk products, and studies have documented that adult consumers have difficulty understanding nicotine concentrations [15]. We decided to experimentally manipulate nicotine content in our study as either “high” or “low” to broadly assess how existing perceptions of nicotine content would influence participants’ responses across reduced-risk products that included or did not include an MRTP claim. Furthermore, grouping products by this nicotine categorization broadly aligns with impending rulemaking on a nicotine product standard for combustible tobacco products.

One methodological approach to assess individual preferences over alternative scenarios that are systematically manipulated is a discrete choice experiment (DCE). This experimental approach has been utilized to assess tobacco product perceptions and estimate behaviors focused on discerning preferences for specific subpopulations such as adolescents [16,17,18] and individuals motivated to quit smoking [19]. As the number of authorized MRTPs in the U.S. grows, a DCE offers an optimal approach to elicit preferences across products based on key attributes (presence vs. absence of MRTP claim).

This study examined relative risk perceptions of four different tobacco products—three products authorized by the FDA to include MRTP claims in their marketing (General Snus Classic White, Philip Morris IQOS, and 22nd Century Group VLN cigarettes) and one not authorized (Juul labs JUUL e-cigarette). All but one product (VLN cigarettes) was commercially available when this study was conducted. In 2021, e-cigarette use (including JUUL products) was the highest among U.S. adults at 4.5% [20], followed by snus at 2.1% [20] and IQOS at 1.1% [21]. Across all products, use is highest among individuals who identify as Non-Latine White, but there is a growing amount of evidence that indicates higher interest in trying IQOS among people of color [22,23]. 

Our study expands on previous research by reporting how products with and without modified risk claims that vary by nicotine content are perceived by a racially and ethnically diverse sample. We aim to examine how products that may pose fewer health risks relative to one another influence perceptions of tobacco product risk, nicotine knowledge, ease of quitting smoking, and intentions to use stratified by claims. Although we hypothesized all demographic groups would perceive MRTP claim-labeled products more favorably than those without claims, we further sought to examine potential group differences. 

## 2. Materials and Methods

### 2.1. Participant Eligibility and Recruitment

Individuals who smoke and do not smoke cigarettes were recruited from July 2022 through October 2022 via the Prolific crowdsourcing platform [24]. Prolific has three screening categorizations for smoking status, “never”, “current”, and “recent”. Using Prolific’s definition, non-smokers in our sample were eligible to participate if they self-reported “never smoking” in Prolific, were between the ages of 21 and 65 years, and smoked fewer than 100 cigarettes in their lifetime. For the smoking sample, Prolific members were eligible to participate if they were between the ages of 21 and 65 years and self-reported “current” or “recent” smoking status in Prolific and reported smoking at least five cigarettes a day. 

Sampling frames for different racial and ethnic demographic groups were created in Prolific using their existing sampling recruitment categories: Non-Hispanic Black or African American, Non-Hispanic White, Non-Hispanic Asians (inclusive of Southeast Asian, East Asian, and South Asian), Latino or Hispanic, and American Indians or Alaskan Natives (AI/AN). Our analyses use the Center for Disease Control’s standard reporting combined format for race and ethnicity: All Individuals, Non-Latine Asian, Non-Latine Native Hawaiian or Pacific Islander, Non-Latine Black, Non-Latine White, Non-Latine AI/AN, Multiracial (compared to monoracial), or Latine [25]. Participants were compensated at USD 12.10 per hour (including survey fees) [26]. All study procedures were approved by the University of Pennsylvania Institutional Review Board (protocol code 851359, 16 May 2022).

### 2.2. Discrete Choice Study Design

Eligible participants were invited to participate in a 30 min survey, which included a consent form, demographic questionnaire, and battery of behavioral and subjective measures and two independent experimental tasks, a discrete choice experiment, and a Maximum Difference (MaxDiff) exercise. We focus on the results of the DCE in the current paper. 

The DCE task was informed by the standard procedures and existing literature [27]. The attributes we tested were nicotine content (high vs. low) and three authorized MRTP products: (1) General Snus Classic White, (2) Philip Morris IQOS, (3) 22nd Century Group VLN cigarette, and (4) one non-MRTP product JUUL e-cigarette. For the task, we used a 2 × 2 × 4 design to determine the eight stimuli conditions (1. Snus low nicotine, 2. Snus high nicotine, 3. JUUL low nicotine, 4. JUUL high nicotine, 5. IQOS low nicotine, 6. IQOS high nicotine, 7. VLN low nicotine, and 8. VLN high nicotine) for two stratifications (Claim vs. No Claim). Stimuli were presented to maximize the information extracted with a minimal number of combinations for 15-choice sets that were stratified by claim [28]. 

Participants were first stratified by claim or no claim (between-subjects) and then randomly shown 15 sets of stimuli with varying nicotine attributes (within-subjects) for the four products (within-subjects). An existing FDA-authorized MRTP claim, or a previously tested claim was used [3]. When an MRTP claim was present, it was not the same across products. This drove the decision to stratify the choice sets by claim with the goal of ruling out the effect of a claim broadly (vs. no claim vs. a different claim). All products included an image and feature information and were presented in the same order for each set (Snus, JUUL, IQOS, VLN). 

### 2.3. Measures

Following Prolific screening eligibility and consent, participants provided demographic information, including age, gender, race, ethnicity, sex assigned at birth, gender identity, sexuality, highest level of education, household income, food and housing security, health care insurance coverage, employment status, perceived social status, and place of birth (U.S.-born vs. foreign-born). For those born outside of the U.S., participants were asked to share how many years they had lived in the U.S. Participants then answered a set of questions on nicotine knowledge, adapted from the Population Assessment of Tobacco and Health (PATH) Study [29] and health system questions such as level of health care system trust [30]. Participants who identified as smokers completed a tobacco use history questionnaire, adult tobacco dependence index [31], and the Fagerstrom Test for Nicotine Dependence (FTND) [32]. 

### 2.4. Primary Outcomes

**Figure 1:**
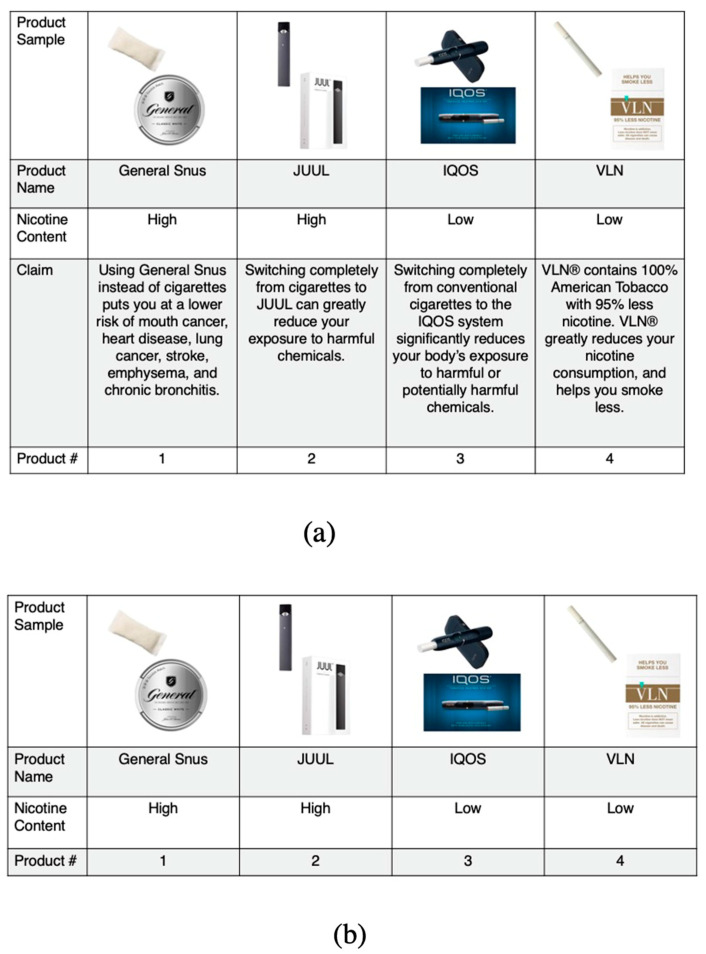
Discrete choice stratified sample choice sets: (**a**) Panel a shows a sample choice set viewed by participants randomized to the modified tobacco product choice sets that included a claim; (**b**) Panel b shows a sample choice set viewed by participants randomized to the modified tobacco product choice sets that did not include a claim.

We tested four outcomes within each choice set. Participants were asked to select one product in relation to the following outcome questions: Which one of these products (1) Would you most like to try? (2) Is the most likely to give you serious disease if you used it regularly? (3) Is least addictive? (4) Would make it easier to quit smoking? Sample choice sets are presented in Figure 1.

### 2.5. Analyses

Data quality procedures were implemented by prohibiting duplicate responses and removing automated responses flagged by Qualtrics features (reCAPTCHA and attention checks) [33]. Attention checks were presented as two questions that asked users to indicate their level of agreement with the following statements: (1) I have never used a computer, tablet, or smartphone, with six response options ranging from strongly disagree to strongly agree, followed by the question, (2) I am currently using a computer, tablet, or smartphone [34]. 

Descriptive statistics were used to characterize the sample. We used multivariable models with generalized estimated equations (GEE) for repeated measures, specifying an exchangeable working correlation structure based on our experimental design. We modeled each outcome independently (most likely to try, most likely to cause serious disease, least addictive, easiest to quit smoking) and dichotomously (choice yes = 1, choice no = 0) both for the full sample and different racial/ethnic groups. The study’s primary aim was to focus on the experimental effects among various racial/ethnic groups. Therefore, to enable greater efficiency and ease of interpretation, analyses used a categorical independent variable reflecting the full set of conditions in the experimental design following an analytical approach used in a previous work [28]. This included the main effects of nicotine level, product type, claim, and their interactions, using the “high nicotine” VLN cigarette with no claim condition as the reference category as it is the product that most closely resembles commercially available cigarettes. We also included covariates for cigarette smoking status, set randomization order, education, age, and sex. Our results are reported as adjusted odds ratios (ORs) and 95% Confidence Intervals for all models. Analyses were conducted in SAS Version 9.4, SAS Institute Inc. (Cary, NC, USA).

## 3. Results

We screened *n* = 1639 individuals for eligibility. Eighty-one (5%) participants were excluded because they did not finish the survey. Furthermore, two participants (0.1%) had a bot score of less than 0.5 based on reCAPTCHA software and 15 (0.9%) participants did not respond to attention checks. The exclusion criteria listed are not mutually exclusive, and individuals who were deemed ineligible did not differ by key demographic characteristics. Our initial intention was to run models for each outcome for each racial and ethnic group separately to allow for comparisons. However, we removed *n* = 68 (<5%) due to small sample sizes and model nonconvergence among Non-Hispanic American Indian, Native American, and Alaska Native (*n* = 20), Native Hawaiian or Other Pacific Islander (*n* = 1), and Multiracial or Other Race (*n* = 47) groups, leaving an analytical sample of *n* = 1484. 

Table 1 reflects our sample characteristics. Participants averaged 34 (SD = 10.6) years of age and were nearly evenly split by sex (51.9% female; 48.0% male; and 0.1% intersex). More than half of participants (56.3%) reported receiving some college education or more, 50.3% reported an annual income of at least $50,000, and over a third, reported current use of combustible cigarettes (36.7%) with an overall mean FTND score of 4.6. Overall, 11.1% of participants were born outside of the U.S. and a total of 364 (23.5%) individuals identified as Non-Latine Black, 283 (18.2%) as Non-Latine Asian, 376 (24.2%) as Latine, and 461 (29.7%) as Non-Latine White. 

### 3.1. Experimental Outcomes

The results of our multivariable logistic regression models stratified by claim for the experimental outcomes are shown in Table 2. We also describe results for all four outcomes among each racial and ethnic group (see Appendix A). Given that we report on the full sample and separate models by race and ethnicity for each outcome, we present results using one categorical variable with all conditions for ease of interpretation and to allow us to comment on patterns across all groups. 

### 3.2. Most Like to Try

Using the full sample, compared to high nicotine VLN cigarettes, participants reported that they were most interested in trying low-nicotine JUUL, regardless of claim: No Claim (OR = 9.4, 95% CI = 7.3, 12.1) and Claim (OR = 6.8, 95% CI = 5.5, 8.3). Participants were least interested in trying high nicotine Classic White Snus, regardless of claim: No Claim (OR = 0.5, 95% CI = 0.4, 0.7) and Claim (OR = 0.5, 95% CI = 0.4, 0.7). Participant’s interest in trying high nicotine VLN with a claim and high nicotine IQOS with a claim or without a claim were no different from the reference group (high nicotine VLN without a claim). All estimates for low-nicotine products were significantly higher from the reference group. No distinct product preference patterns emerged for this outcome based on claims. 

### 3.3. Serious Disease If Used Regularly

For participants viewing a claim, products that were high nicotine were perceived as being more likely to cause serious disease relative to low nicotine products [(VLN OR = 0.8, 95% CI = 0.7, 0.9), (Snus OR = 0.7, 95% CI = 0.6, 0.9), (JUUL OR = 0.4, 95% CI = 0.3, 0.5)]. Yet, the addition of a claim significantly decreased participants’ perceived risk of serious disease for VLN cigarettes by 20% (OR = 0.8, 95% CI = 0.7, 0.9). Overall, the perceived odds of serious disease were least likely for low nicotine products compared to high nicotine products. For example, low nicotine IQOS was perceived as the product least likely to cause serious disease, regardless of claim: No Claim (OR = 0.1, 95% CI = 0.04, 0.1) and Claim (OR = 0.1, 95% CI = 0.1, 0.1). Stated another way, regardless of claim, a low nicotine IQOS product was perceived as 90% less likely to cause serious disease compared to high nicotine VLN without a claim. Lastly, participants perceived high nicotine Classic White Snus, without a claim, to be just as likely to cause serious disease as the reference group (high nicotine VLN without a claim).

### 3.4. Least Addictive

Low nicotine products were perceived as being less addictive than high nicotine products. For example, individuals ranked low nicotine JUUL without a claim as the product they perceived as least addictive (OR = 18.1, 95% CI = 14.3, 23.0), followed by low nicotine VLN cigarettes with a claim (OR = 15.6, 95% CI = 2.5, 19.4). There was no consistent pattern concerning claims on perceived addictiveness.

### 3.5. Easier to Quit Smoking

Low nicotine JUUL without a claim (OR = 14.7, 95% CI = 11.5, 18.7) was endorsed most often for making it easier to quit smoking. Notably, the magnitude of the estimates for low nicotine JUUL without a claim (OR = 14.7, 95% CI = 11.5, 18.7) and low nicotine VLN cigarettes with a claim (OR = 11.6, 95% CI = 9.3, 14.5) were higher relative to other possible combinations in the whole sample. Every combination of low nicotine products was perceived as more likely to facilitate quitting compared to high nicotine products. There was no clear pattern for how claims impacted participants’ preferences for this outcome.

### 3.6. Group Differences by Race and Ethnicity

Tables for all outcomes by race/ethnicity are shown in Appendix A. Overall, across all four outcomes, patterns appeared similar between different racial and ethnic groups as compared to the full sample. However, there were some exceptions. For example, similar to the overall sample, Non-Latine Asians were most interested in trying low-nicotine IQOS, regardless of claim: No Claim (OR = 24.0, 95% CI 13.1, 43.8) and Claim (OR = 15.4, 95% CI 9.5, 25.1). However, estimates for this product were two-fold higher for Non-Latine Asians than Non-Latine Black individuals and Latine individuals and four-fold higher than Non-Latine White individuals.

For the least addictive outcome, like the overall sample, Non-Latine Black individuals ranked low nicotine JUUL with no claim as least addictive. However, when comparing this estimate to other racial and ethnic demographic groups, the order of magnitude was half as much (OR = 11.0, 95% CI = 7.0, 17.4) compared to the estimates for Non-Latine Asians, Latines, and Non-Latine White individuals. Another important distinction for this outcome was that Non-Latine Black individuals perceived high nicotine products as no different in addictiveness from the reference group (high nicotine VLN cigarettes without a claim), except for one product, high nicotine VLN cigarettes with a claim (OR = 3.5, 95% CI = 2.3, 5.4). 

Finally, like the overall sample, Non-Latine Asians ranked low-nicotine JUUL without a claim as the product with the highest odds of facilitating quitting smoking. However, the estimate for this product was much higher (OR = 21.3, 95% CI = 11.5, 39.4) compared Non-Latine Black (OR = 11.2, 95% CI = 7.0, 18.1), Non-Latine White (OR = 14.8, 95% CI = 9.5, 22.9), and Latine (OR = 14.6, 95% CI = 9.2, 23.2) individuals. Perceptions differed between racial and ethnic groups for the product participants perceived would be most helpful to quit smoking. For Non-Latine Asian and Non-Latine White individuals, the odds ratio was highest for low nicotine JUUL without a claim [(Non-Latine Asians OR = 21.3, 95% CI = 11.5, 39.4), (Non-Latine White individuals, 14.8, 95% CI = 9.5, 22.9)]. In comparison, low nicotine VLN cigarettes with a claim were the product with the highest odds for Non-Latine Black (OR = 11.6, 95% CI = 7.5, 17.8) and Latine (OR = 14.7, 95% CI = 9.7, 22.4) individuals.

## 4. Discussion

This study is among the first to explore intentions to try, the perceived harm of, the addictiveness of, and the ease of quitting of tobacco products that span a continuum of harm [35] in a racially and ethnically diverse sample. Results broadly demonstrate that low nicotine products were preferred over high nicotine products across outcomes. Participants were most interested in trying low nicotine JUUL regardless of claims, and perceived low nicotine IQOS as the product least likely to cause serious disease if used regularly, regardless of claims. Data patterns suggest that participants broadly perceived low nicotine products as less addictive than high nicotine products, and perceived low nicotine JUUL without a claim as the least addictive product. Participants reported that low nicotine products without a claim would make it easier to quit smoking. Findings indicate that result patterns were largely consistent across race/ethnic groups models. 

Research suggests that MRTP claims have a limited impact on product switching and perceptions of harm reduction [36]. For the perceived risk of serious disease outcome, the inclusion of an MRTP claim significantly decreased the perceived risk of serious disease for all products except high nicotine snus. This could, in part, be driven by product misperceptions about snus being as harmful or more harmful than smoking [3] or an overall lack of awareness of the relative harms of smokeless tobacco products compared to cigarettes [37]. A recent study found that participants thought snus with an MRTP claim was less safe and led to higher chemical exposure than HTPs and e-cigarettes with an MRTP claim [38].

When we consider the interaction between MRTP claim and nicotine content, high nicotine products that include a claim were perceived as being more likely to cause serious disease relative to low nicotine products with a claim. While we want products that include a claim to show that they confer less risk of disease in order to more accurately convey reduced disease risk, findings suggest that MRTP messages may require additional nicotine-specific messaging, especially among products that emphasize nicotine content in their marketing. This differs from existing research on MRTP claims that identify issues of credibility related to claims made by for-profit companies [39] or potentially conflicting information with the presence of a government warning label [40]. Similarly, participants endorsed high nicotine products as more addictive than low nicotine products overall. However, within these groupings, participants do not necessarily distinguish consistently by product or claim, suggesting that additional messaging may be necessary to help individuals understand differences in addictiveness when comparing modified-risk tobacco products. 

A few important distinctions emerged when comparing outcome preferences among racial and ethnic demographic groups, including the strong preference to try low nicotine IQOS among Non-Latine Asians and a high perception that low nicotine JUUL without a claim would make it easier to quit smoking. Greater intentions to try IQOS among this racial group is concerning as IQOS use has been correlated with identifying as Asian or Hispanic in the U.S. [21]. While this pattern was found in a cross-sectional survey, it will be important to monitor uptake in this demographic group over time, as Phillip Morris International has plans to resume the sale of IQOS in the U.S. in 2023 [41]. Evidence from Korea has shown that tobacco companies have facilitated rapid uptake of HTPs by marketing their products as lower in harm and as an aid to quitting smoking. Our results suggest that Non-Latine Asians do not perceive this product to be as helpful in facilitating quitting compared to JUUL and VLN. Park et al. caution whether HTPs should be considered as cessation aids because a significant proportion of dual product users report low intentions to quit cigarettes [42]. This is important from a health communication perspective, as many of the health benefits of MRTPs are contingent on switching completely from cigarettes [43]. 

For the addictiveness outcome, Non-Latine Black individuals perceived high nicotine products as no different from the reference group, apart from one product, high nicotine VLN cigarettes with a claim. This pattern of results may reflect previously reported patterns of higher absolute harm perceptions among Non-Latine Black individuals compared to other racial and ethnic groups for products such as smokeless tobacco, e-cigarettes, cigars, hookah, and pipe tobacco [44]. While our findings indicate that this demographic group may be more averse to high nicotine products overall, there is little indication that the inclusion of a claim helped participants rank these products on addictiveness. Additional information to clarify the connection between reduced risk or exposure claims across products may be particularly useful for this racial group, such as the inclusion of potentially harmful constituents.

Our study provides an initial picture of how participants perceived tobacco products varying in nicotine content and MRTP claim in a racially and ethnically diverse sample. By looking at racial and ethnic demographic subgroups, we were able to tease out small but important differences across our outcomes. We did not control for marketing exposure or current use of products, which is a limitation. We also are unable to quantify exactly how participants distinguish between low and high nicotine; however, the purpose of this study was to broadly understand how individuals perceive different tobacco products that vary by nicotine content. Although not all participants were exposed to the high nicotine VLN condition, a high nicotine VLN cigarette without a health claim was selected as the reference group because it was the experimental manipulation that most resembled a non-MRTP approved commercially available cigarette on the U.S. market. 

At the time that these data were collected, VLN cigarettes were not commercially available in the U.S. The product has since been approved for distribution in Texas, California, and Florida [45], which will likely have implications for how a product that is inherently low nicotine is marketed and perceived in the future. Although our design included a stratification for claims, which limited its interpretation as a between-subject effect, we felt this was appropriate, provided the varied content of the claims. For example, the claims’ content did not consistently address one theme (e.g., relative to cigarettes, nicotine content, chemical exposure), so we stratified conditions by claims for higher internal validity in determining the effects of a claim (vs. no claim) as opposed to the potential bias driven by the content of different claims, as studied in other DCE work [46]. Experimental work should study the effects of specific claim content and themes further.

Considering our limitations, there was limited indication that claims were driving product perceptions except for the perceived risk of serious disease outcome. This is contrary to findings in a previously completed study that evaluated the impact of MRTP claims for HTPs, electronic cigarettes, and snus, which found that modified risk and exposure claims led individuals to perceive these products as being lower in chemical quantities and lower in harm [38]. Our results suggest that claims do not appear to be driving perceptions in the current sample for addictiveness, intention to use, or ease of quitting, despite the inclusion of reduced risk/exposure claims contrasted alongside other modified risk products. These findings underscore that reduced exposure/risk claims remain a source of confusion for participants when taken into consideration with other factors (nicotine content and products). 

## 5. Conclusions

In a racially and ethnically diverse sample, participants were asked to compare tobacco products that vary by nicotine content and modified risk claims for behavior-related outcomes. Overall, stated preferences for intention to try were highest for low nicotine JUUL regardless of a claim, and participants perceived low nicotine IQOS without a claim to be the least likely to cause serious disease. Participants perceived low nicotine products as less addictive than high nicotine products, with low nicotine JUUL without a claim as the product that was least addictive and most likely to facilitate quitting. There was a strong preference to try low nicotine IQOS among Non-Latine Asians and a high perception that low nicotine JUUL without a claim would most facilitate quitting smoking. Non-Latine Black individuals perceived high nicotine (JUUL, IQOS, and Classic White Snus) to be just as addictive as the high nicotine VLN cigarettes without a claim. Overall, results show product type and nicotine content were primary drivers of perceptions among different racial and ethnic groups, suggesting that regulatory efforts should be guided by our understanding of how MRTP claims interact with different product characteristics.

## Figures and Tables

**Table 1:** Full sample demographic characteristics, *n* = 1484.

Age	34.2 (10.6)
Sex	
Female	771 (51.9%)
Male	712 (47.9%)
Intersex	1 (0.1%)
Race/Ethnicity	
Non-Latine Black	364 (23.5%)
Non-Latine Asian	283 (18.2%)
Non-Latine White	461 (29.7%)
Latine	376 (24.2%)
Foreign Born	
Yes	165 (11.1%)
Education ^1^	
GED or less	14 (14.4%)
Some Technical School	433 (29.2)
College of Beyond	836 (56.3%)
Income ^2^	
More than 50,000	746 (50.3%)
Smoking Status	
Current Smoker	544 (36.7%)
Non-Smoker	940 (63.3%)

Note. Data expressed as Mean (SD) or N (%). ^1^ One participant did not report education. ^2^ One participant did not report income.

**Table 2:** Experimental results (all individuals *n* = 1484).

Conditions	Most Like to Try	Serious Disease If Used Regularly	Least Addictive	Easier to Quit Smoking
	Odds Ratios (CI)	Odds Ratios (CI)	Odds Ratios (CI)	Odds Ratios (CI)
No Claim, High nicotine, VLN	Ref.	Ref.	Ref.	Ref.
Claim, High nicotine, VLN	1.1 (0.8, 1.3)	**0.8 (0.7, 0.9)**	**3.8 (3.0, 4.8)**	**3.7 (2.9, 4.6)**
No Claim, High nicotine, JUUL	**2.2 (1.7, 2.7)**	**0.3 (0.3, 0.4)**	**2.0 (1.6, 2.6)**	**2.5 (1.9, 3.1)**
Claim, High nicotine, JUUL	**2.2 (1.8, 2.8)**	**0.4 (0.3, 0.5)**	**2.0 (1.5, 2.5)**	**2.3 (1.8, 3.0)**
No Claim, High nicotine, IQOS	0.9 (0.7, 1.2)	**0.3 (0.3, 0.4)**	**1.3 (1.0, 1.7)**	1.1 (0.9, 1.5)
Claim, High nicotine, IQOS	1.2 (1.0, 1.5)	**0.3 (0.2, 0.3)**	**1.8 (1.4, 2.3)**	**1.5 (1.2, 1.9)**
No Claim, High nicotine, Snus	**0.5 (0.4, 0.7)**	0.9 (0.7, 1.1)	**1.8 (1.4, 2.3)**	**1.9 (1.4, 2.4)**
Claim, High nicotine, Snus	**0.5 (0.4, 0.7)**	**0.7 (0.6, 0.9)**	**1.6 (1.2, 2.0)**	1.3 (1.0, 1.7)
No Claim, Low nicotine, VLN	**3.5 (3.0, 4.0)**	**0.3 (0.3, 0.3)**	**8.9 (7.5, 10.5)**	**6.2 (5.2, 7.3)**
Claim, Low nicotine, VLN	**3.7 (3.0, 4.6)**	**0.3 (0.3, 0.4)**	**15.6 (12.5, 19.4)**	**11.6 (9.3, 14.5)**
No Claim, Low nicotine, JUUL	**9.4 (7.3, 12.1)**	**0.1 (0.1, 0.1)**	**18.1 (14.3, 23.0)**	**14.7 (11.5, 18.7)**
Claim, Low nicotine, JUUL	**6.8 (5.5, 8.3)**	**0.2 (0.1, 0.2)**	**9.9 (8.0, 12.2)**	**7.7 (6.2, 9.6)**
No Claim, Low nicotine, IQOS	**4.6 (3.7, 5.7)**	**0.1 (0.0, 0.1)**	**13.5 (10.8, 16.8)**	**9.0 (7.1, 11.2)**
Claim, Low nicotine, IQOS	**4.9 (4.0, 5.9)**	**0.1 (0.1, 0.1)**	**11.4 (9.2, 14.0)**	**7.7 (6.2, 9.5)**
No Claim, Low nicotine, Snus	**2.0 (1.5, 2.5)**	**0.3 (0.2, 0.3)**	**11.6 (9.2, 14.8)**	**7.6 (6.0, 9.6)**
Claim, Low nicotine, Snus	**2.8 (1.8, 2.7)**	**0.3 (0.3, 0.4)**	**8.1 (6.5, 10.1)**	**5.2 (4.1, 6.5)**

Bold = Statistically significant, *p* < 0.05.

## Data Availability

Data available upon request.

## Appendix A

**Table A1:** Experimental results (Non-Latine Black *n* = 364).

Conditions	Most Like to Try	Serious Disease If Used Regularly	Least Addictive	Easier to Quit Smoking
	Odds Ratios (CI)	Odds Ratios (CI)	Odds Ratios (CI)	Odds Ratios (CI)
Claim				
High nicotine, VLN	1.2 (0.8, 2.0)	0.8 (0.6, 1.0)	**3.5 (2.3, 5.4)**	**3.8 (2.5, 6.0)**
Low nicotine, VLN	**5.3 (3.5, 8.2)**	**0.3 (0.2, 0.4)**	**12.9 (8.5, 19.4)**	**11.6 (7.5, 17.8)**
High nicotine, JUUL	**2.3 (1.5, 3.7)**	**0.6 (0.4, 0.8)**	1.6 (1.0, 2.5)	**1.8 (1.1, 2.9)**
Low nicotine, JUUL	**7.4 (4.9, 11.4)**	**0.2 (0.1, 0.3)**	**6.2 (4.1, 9.2)**	**5.6 (3.7, 8.6)**
High nicotine, IQOS	1.4 (0.9, 2.3)	**0.4 (0.3, 0.6)**	1.5 (0.9, 2.4)	1.4 (0.9, 2.3)
Low nicotine, IQOS	**6.2 (4.0, 9.4)**	**0.1 (0.1, 0.2)**	**6.7 (4.5, 10.0)**	**5.9 (3.9, 8.9)**
High nicotine, Snus	0.7 (0.4, 1.2)	0.9 (0.6, 1.2)	1.0 (0.6, 1.7)	1.2 (0.7, 2.0)
Low nicotine, Snus	**3.0 (2.0, 4.7)**	**0.3 (0.2, 0.4)**	**4.5 (3.0, 6.8)**	**4.8 (3.2, 7.4)**
No Claim				
High nicotine, VLN	Ref.	Ref.	Ref.	Ref.
Low nicotine, VLN	**3.7 (2.6, 5.1)**	**0.2 (0.2, 0.3)**	**6.6 (4.8, 9.0)**	**5.3 (3.9, 7.2)**
High nicotine, JUUL	**2.5 (1.5, 4.0)**	**0.4 (0.3, 0.6)**	1.5 (0.9, 2.4)	**2.2 (1.3, 3.6)**
Low nicotine, JUUL	**10.1 (6.1, 16.8)**	**0.1 (0.1, 0.2)**	**11.0 (7.0, 17.4)**	**11.2 (7.0, 18.1)**
High nicotine, IQOS	1.1 (0.7, 1.8)	**0.5 (0.4, 0.7)**	1.1 (0.7, 1.7)	1.1 (0.7, 1.9)
Low nicotine, IQOS	**5.8 (3.6, 9.2)**	**0.1 (0.1, 0.1)**	**8.8 (5.7, 13.6)**	**7.5 (4.8, 11.8)**
High nicotine, Snus	1.1 (0.6, 1.9)	1.1 (0.8, 1.6)	1.3 (0.8, 2.1)	**1.8 (1.1, 3.0)**
Low nicotine, Snus	**3.1 (1.9, 5.1)**	**0.3 (0.2, 0.4)**	**7.6 (4.8, 12.0)**	**6.8 (4.3, 10.9)**

Bold = Statistically significant, *p* < 0.05.

**Table A2:** Experimental results (Non-Latine Asian *n* = 283).

Conditions	Most Like to Try	Serious Disease If Used Regularly	Least Addictive	Easier to Quit Smoking
	Odds Ratios (CI)	Odds Ratios (CI)	Odds Ratios (CI)	Odds Ratios (CI)
Claim				
High nicotine, VLN	1.1 (0.6, 2.1)	0.8 (0.5, 1.1)	**3.2 (1.8, 5.8)**	**2.9 (1.6, 5.3)**
Low nicotine, VLN	**6.4 (3.9, 10.6)**	**0.3 (0.2, 0.5)**	**16.2 (9.3, 28.3)**	**11.5 (6.62, 19.82)**
High nicotine, JUUL	**3.1 (1.8, 5.2)**	**0.3 (0.2, 0.5)**	**2.0 (1.1, 3.7)**	**2.2 (1.2, 4.1)**
Low nicotine, JUUL	**15.4 (9.5, 25.1)**	**0.1 (0.1, 0.2)**	**13.5 (7.8, 23.4)**	**11.6 (6.8, 19.9)**
High nicotine, IQOS	1.2 (0.7, 2.1)	**0.2 (0.2, 0.3)**	1.2 (0.7, 2.2)	0.9 (0.5, 1.6)
Low nicotine, IQOS	**9.0 (5.6, 14.5)**	**0.1 (0.0, 0.1)**	**13.3 (7.8, 22.7)**	**10.1 (6.0, 17.1)**
High nicotine, Snus	0.7 (0.4, 1.4)	**0.5 (0.4, 0.7)**	1.7 (0.9, 3.3)	1.5 (0.8, 2.9)
Low nicotine, Snus	**6.1 (3.7, 10.1)**	**0.1 (0.1, 0.2)**	**13.4 (7.7, 23.2)**	**9.9 (5.8, 17.1)**
No Claim				
High nicotine, VLN	Ref.	Ref.	Ref.	Ref.
Low nicotine, VLN	**4.6 (3.2, 6.7)**	**0.2 (0.2, 0.3)**	**9.1 (5.8, 14.2)**	**6.2 (4.1, 9.4)**
High nicotine, JUUL	**3.7 (2.1, 6.3)**	**0.2 (0.2, 0.4)**	**2.4 (1.3, 4.5)**	**2.7 (1.5, 5.0)**
Low nicotine, JUUL	**24.0 (13.1, 43.8)**	**0.1 (0.0, 0.1)**	**24.9 (13.5, 45.9)**	**21.3 (11.5, 39.4)**
High nicotine, IQOS	1.5 (0.8, 2.6)	**0.3 (0.2, 0.4)**	1.4 (0.8, 2.5)	1.2 (0.6, 2.2)
Low nicotine, IQOS	**7.8 (4.6, 13.0)**	**0.1 (0.0, 0.1)**	**13.1 (7.5, 22.9)**	**9.8 (5.6, 17.1)**
High nicotine, Snus	0.7 (0.4, 1.4)	**0.6 (0.4, 1.0)**	1.6 (0.8, 3.1)	1.7 (0.9, 3.3)
Low nicotine, Snus	**4.6 (2.7, 8.0)**	**0.1 (0.1, 0.2)**	**16.5 (9.0, 30.1)**	**11.5 (6.3, 20.9)**

Bold = Statistically significant, *p* < 0.05.

**Table A3:** Experimental results (Latine *n* = 376).

Conditions	Most Like to Try	Serious Disease If Used Regularly	Least Addictive	Easier to Quit Smoking
	Odds Ratios (CI)	Odds Ratios (CI)	Odds Ratios (CI)	Odds Ratios (CI)
Claim				
High nicotine, VLN	1.3 (0.9, 2.1)	**0.7 (0.5, 1.0)**	**5.2 (3.3, 8.1)**	**4.5 (2.9, 7.0)**
Low nicotine, VLN	**4.0 (2.7, 6.0)**	**0.4 (0.2, 0.5)**	**21.1 (13.8, 32.3)**	**14.7 (9.7, 22.4)**
High nicotine, JUUL	**2.6 (1.7, 4.0)**	**0.4 (0.3, 0.5)**	**2.2 (1.4, 3.4)**	**2.6 (1.7, 4.2)**
Low nicotine, JUUL	**7.7 (5.3, 11.3)**	**0.2 (0.1, 0.2)**	**11.0 (7.3, 16.6)**	**8.0 (5.3, 12.0)**
High nicotine, IQOS	1.2 (0.7, 1.8)	**0.3 (0.2, 0.3)**	**2.2 (1.4, 3.6)**	1.4 (0.9, 2.3)
Low nicotine, IQOS	**4.4 (3.0, 6.4)**	**0.1 (0.1, 0.1)**	**13.2 (8.8, 19.8)**	**7.6 (5.1, 11.4)**
High nicotine, Snus	0.8 (0.5, 1.3)	**0.7 (0.5, 0.9)**	**1.7 (1.0, 3.0)**	1.2 (0.7, 1.9)
Low nicotine, Snus	**2.6 (1.7, 3.9)**	**0.4 (0.3, 0.5)**	**9.3 (6.0, 14.4)**	**4.9 (3.1, 7.5)**
No Claim				
High nicotine, VLN	Ref.	Ref.	Ref.	Ref.
Low nicotine, VLN	**4.8 (3.6, 6.4)**	**0.3 (0.2, 0.4)**	**10.9 (8.0, 15.0)**	**7.3 (5.4, 10.0)**
High nicotine, JUUL	**2.3 (1.5, 3.5)**	**0.4 (0.3, 0.5)**	**2.3 (1.4, 3.7)**	**1.9 (1.2, 3.1)**
Low nicotine, JUUL	**11.2 (7.1, 17.7)**	**0.1 (0.0, 0.1)**	**20.8 (13.2, 32.9)**	**14.6 (9.2, 23.2)**
High nicotine, IQOS	0.9 (0.6, 1.4)	**0.3 (0.2, 0.5)**	1.6 (0.9, 2.6)	1.3 (0.8, 2.0)
Low nicotine, IQOS	**5.1 (3.4, 7.6)**	**0.1 (0.0, 0.1)**	**17.6 (11.6, 26.8)**	**10.0 (6.5, 15.1)**
High nicotine, Snus	**0.4 (0.2, 0.7)**	0.9 (0.6, 1.3)	**2.5 (1.5, 4.1)**	**2.2 (1.4, 3.6)**
Low nicotine, Snus	**2.1 (1.3, 3.2)**	**0.3 (0.2, 0.4)**	**13.4 (8.4, 21.4)**	**8.3 (5.3, 13.2)**

Bold = Statistically significant, *p* < 0.05.

**Table A4:** Experimental results (Non-Latine White *n* = 461).

Conditions	Most Like to Try	Serious Disease If Used Regularly	Least Addictive	Easier to Quit Smoking
	Odds Ratios (CI)	Odds Ratios (CI)	Odds Ratios (CI)	Odds Ratios (CI)
Claim				
High nicotine, VLN	0.8 (0.5, 1.2)	0.8 (0.6, 1.1)	**3.8 (2.5, 5.7)**	**3.4 (2.3, 5.2)**
Low nicotine, VLN	**2.2 (1.5, 3.1)**	**0.4 (0.3, 0.6)**	**15.1 (10.4, 22.0)**	**9.9 (6.6, 14.8)**
High nicotine, JUUL	**1.8 (1.2, 2.6)**	**0.3 (0.2, 0.4)**	**2.3 (1.5, 3.6)**	**2.6 (1.7, 4.1)**
Low nicotine, JUUL	**4.0 (2.8, 5.7)**	**0.2 (0.1, 0.2)**	**11.6 (8.0, 16.9)**	**7.5 (5.1, 11.1)**
High nicotine, IQOS	1.2 (0.8, 1.7)	**0.2 (0.1, 0.3)**	**2.2 (1.4, 3.4)**	**2.0 (1.3, 3.1)**
Low nicotine, IQOS	**3.5 (2.4, 5.0)**	**0.1 (0.0, 0.1)**	**15.1 (10.5, 21.7)**	**8.3 (5.6, 12.2)**
High nicotine, Snus	**0.3 (0.2, 0.5)**	0.8 (0.6, 1.1)	**2.1 (1.3, 3.3)**	1.4 (0.9, 2.3)
Low nicotine, Snus	0.8 (0.5, 1.2)	**0.5 (0.3, 0.6)**	**9.0 (6.1, 13.1)**	**3.7 (2.5, 5.7)**
No Claim				
High nicotine, VLN	Ref.	Ref.	Ref.	Ref.
Low nicotine, VLN	**2.4 (1.9, 3.1)**	**0.4 (0.3, 0.5)**	**10.1 (7.6, 13.4)**	**6.1 (4.5, 8.3)**
High nicotine, JUUL	**1.6 (1.0, 2.4)**	**0.3 (0.2, 0.5)**	**2.3 (1.5, 3.6)**	**3.0 (1.9, 4.7)**
Low nicotine, JUUL	**5.2 (3.3, 8.0)**	**0.1 (0.1, 0.20)**	**21.3 (14.1, 32.2)**	**14.8 (9.5, 22.9)**
High nicotine, IQOS	0.7 (0.5, 1.1)	**0.3 (0.2, 0.4)**	1.3 (0.9, 2.0)	1.0 (0.7, 1.6)
Low nicotine, IQOS	**3.0 (2.0, 4.4)**	**0.0 (0.0, 0.1)**	**16.7 (11.4, 24.6)**	**9.1 (6.0, 13.7)**
High nicotine, Snus	**0.3 (0.2, 0.6)**	0.9 (0.6, 1.3)	**2.0 (1.3, 3.2)**	**1.7 (1.0, 2.8)**
Low nicotine, Snus	0.8 (0.5, 1.3)	**0.3 (0.2, 0.5)**	**12.8 (8.5, 19.1)**	**5.9 (3.8, 9.1)**

Bold = Statistically significant, *p* < 0.05.
